# Do reminders of the crime reverse the memory-undermining effect of simulating amnesia?

**DOI:** 10.3758/s13421-019-00939-z

**Published:** 2019-05-17

**Authors:** I. Mangiulli, T. Lanciano, K. van Oorsouw, M. Jelicic, A. Curci

**Affiliations:** 1 Department of Education, Psychology, Communication, University of Bari A. Moro, Bari, Italy; 2 Forensic Psychology Section, Faculty of Psychology and Neuroscience, Maastricht University, P.O. Box 616, 6200 MD Maastricht, The Netherlands

**Keywords:** Crime-related amnesia, Simulating, Memory loss, Rehearsal, Reminders

## Abstract

Research shows that simulating amnesia impairs actual memory for a mock crime. Lack of rehearsal has been suggested as the most likely explanation for this finding because feigning amnesia is linked to reduced thinking about the offence. We investigated whether reminders about the crime could reverse the memory-undermining effect of simulation. In two studies, participants watched a video of a violent crime. After, they were asked to either simulate amnesia or confess the crime. During the week between the first and second memory test phase, participants were provided with reminders of the crime in two different modalities. In Study 1 (pilot), participants received frames of the mock crime video via WhatsApp. Findings showed that such reminders did not enhance ex-simulators’ memory. In Study 2, participants were asked to put sequences of the mock crime in the right order. This latter modality led to enhanced memory for the offence in simulating participants. Theoretical and practical implications of our findings for the legal field are discussed.

**Electronic supplementary material:**

The online version of this article (10.3758/s13421-019-00939-z) contains supplementary material, which is available to authorized users.

There is debate about the existence of bona fide crime-related amnesia. While some authors argue that crime-related amnesia is due to a mismatch in an offender’s emotional state between the time of the crime and the moment he or she is interrogated by the police (e.g., Bourget & Whitehurst, 2007; Kopelman, 1995; Porter, Birt, Yuille, & Hervé, 2001), others reason that crime-related amnesia is an improbable outcome (e.g., Christianson & Merckelbach, 2004), because people usually are well able to remember their own actions (action superiority effect; Engelkamp & Zimmer, 1994; McGaugh, 2006). So far, the veracity of crime-related amnesia claims without an organic cause has been often queried, rendering fabrication of such a condition (i.e., malingering) a possible explanation as to why a person would claim memory loss after committing a crime (Jelicic, 2018; Jelicic & Merckelbach, 2007; Jelicic, Merckelbach, & Boskovic, 2017). Amnesia is frequently reported by people who committed violent and passionate crimes (Pyszora, Fahy, & Kopelman, 2014). Note that 29% of a 1-year cohort of people sentenced to life imprisonment claimed amnesia surrounding their crime (31.4% of those convicted of homicide; Pyszora, Barker, & Kopelman, 2003). Many criminal offenders, indeed, claim memory loss for their deed in order to try to obstruct police investigation and/or interfere with the trial proceedings (Cima, Merckelbach, Nijman, Knauer, & Hollnack, 2002; Tysse, 2005; Tysse & Hafemeister, 2006).

When defendants claim memory loss surrounding their criminal act, crucial crime-related information might be forgotten. Several studies (e.g., Bylin & Christianson, 2002; Christianson & Bylin 1999; Mangiulli, Van Oorsouw, Curci, Merckelbach, & Jelicic, 2018; Romeo, Otgaar, Smeets, Landström, & Jelicic, 2018; Van Oorsouw & Merckelbach, 2004, 2006) observed that when 1 week later participants were asked to genuinely account for the crime experience, individuals who had initially been instructed to feign amnesia for the same target event (i.e., simulators) had poorer memories than those who were instructed to truthfully recall the crime both times (i.e., confessors). This effect is known as the memory-undermining effect of simulated crime-related amnesia (e.g., Mangiulli et al., 2018; Van Oorsouw & Merckelbach, 2004). Relatedly, research shows that participants initially asked to pretend memory loss for the criminal experience increased the number of memory errors over time (e.g., Mangiulli et al., 2018; Van Oorsouw & Giesbrecht, 2008). Albeit crime-related amnesia claims might mostly lead to omissions (e.g., Christianson & Bylin, 1999; Van Oorsouw & Merckelbach, 2004), previous studies showed that the act of feigning amnesia might even imply withholding some crucial crime-related details while distorting and/or fabricating new information on the initial memory test (Bylin & Christianson, 2002; Van Oorsouw & Merckelbach, 2004). Thus, it is quite common that feigning participants report more errors over time as compared with genuinely responding participants (i.e., confessors).

To test the memory-undermining effect of feigning amnesia, some studies included a control condition (e.g., Bylin & Christianson, 2002; Mangiulli et al., 2018; Sun, Punjabi, Greenberg, & Seamon, 2009; Van Oorsouw & Merckelbach, 2004). On the delayed recall, participants who had initially feigned amnesia performed similar to the delayed testing-only group (i.e., controls). Given that both simulators and controls did not actually rehearse the crime as compared with confessors during the first memory phase, this lack of rehearsal might explain the memory-undermining effect in simulating participants. Yet, Sun et al. (2009) argued that “there is no feigned amnesia effect other than differential practice at recall” (p. 89). In other words, because simulators—equally to controls—were given one less occasions to recall the crime, the memory-undermining effect of feigning amnesia would be nothing more than a mere differential practice at recall rather than a detrimental mnemonic effect due to the act of simulating memory loss for a crime.

However, one could argue that in a real-life situation, offenders who committed a crime may have several opportunities to “rehearse” the criminal experience. They might think, for instance, about how they came to commit their criminal acts, how they could have prevented the offence, or perhaps even develop unwanted intrusions or flashbacks of the crime (e.g., Evans, 2006; Evans, Ehlers, Mezey, & Clark, 2007a, 2007b). Generally speaking, rehearsal can enhance memory consolidation (Anderson, Bjork, & Bjork, 1994; Glover, 1989), improving memory for facts, such as spatial and temporal details, and activity components of the events (Marsh, Tversky, & Hutson, 2005). Furthermore, offenders who claimed amnesia surrounding their crime might even individually rehearse what they experienced in order to be consistent with their feigned story during police interrogations. It has been shown that such (mental) practice has a positive and significant effect on memory performance (e.g., Driskell, Copper, & Moran, 1994; see also the testing effect, Roediger & Karpicke, 2006). Consequently, this “self-rehearsal practice” might prevent offenders from easily forgetting the crime.

## Overview and hypotheses

We aimed to investigate whether simulators who are prompted to think about the crime no longer demonstrate the memory-undermining effect of simulating amnesia. We showed participants a mock crime video and asked them to either feign amnesia (simulator group) or to confess (confessor group) when asked about their involvement in the crime during a first memory test phase (T1). Additionally, in both studies, a delayed-testing only group (i.e., controls) was included to empirically test the lack of rehearsal effect. Next, we provided half of the participants with either frames (Study 1; pilot) or written sequences of the mock crime video (Study 2). Finally, each participant was asked to genuinely account for the mock crime event. We anticipated that simulating amnesia would undermine memory for the mock crime in absence of reminders (i.e., simulating amnesia effect; Hypothesis 1). However, we expected that reminders of the crime would increase simulators’ performance over time, leading ex-simulators to recollecting as many correct details as confessors (Hypothesis 2). We also predicted that both confessors and simulators who were given reminders to rehearse would recall more correct crime-related details than a delayed-testing only control group (Hypothesis 3). Finally, we expected that simulators would make more errors than confessors at the second memory test phase irrespective of the rehearsal induction (Hypothesis 4).

## Study 1 (pilot)

We conducted a pilot study to examine whether rehearsal would reverse the memory-undermining effect of simulated amnesia. Based on previous research (e.g., Sun et al., 2009; Van Oorsouw & Merckelbach, 2004), we designed this study with an overall sample size that would have been sufficient to detect the effect of our main hypothesis (i.e., simulating amnesia effect; Hypothesis 1).

### Method

#### Participants and design

A total of 90 undergraduate students (65% women; *M*_age_ = 22.58 years, *SD* = 3.19) at the University of Bari were recruited using advertisements and a snowballing sampling technique (Goodman, 1961). Participants were rewarded with 1 course-credit hour. They were evenly distributed over the three conditions—simulators (*N* = 30), confessors (*N* = 30), and controls (*N* = 30). The study adopted a 3 (condition: simulators vs. confessors vs. controls) × 2 (rehearsal induction: reminder vs. no reminder) between-subjects design. The dependent variable was the correctness of memory calculated as free-recall and cued-recall scores. Additionally, we calculated free and cued error scores.

#### Measures and procedure

The study consisted of two phases, both conducted in a quiet test room. Participants were randomly assigned to one of three conditions. First, each participant was asked to fill out the Structured Inventory of Malingered Symptomatology (SIMS; Smith & Burger, 1997) in order to exclude individual differences in terms of malingering tendency before the experimental phase. The SIMS^1^ is a self-report screening instrument for malingering and consists of 75-item true–false questions. It contains bizarre items that pertain to bizarre or atypical symptoms (e.g. “Sometimes my muscles go limp for no apparent reason so that my arms and legs feel as though they weigh a ton”). The items are divided into five subscales (Affective Disorders; Amnestic Disorders; Low Intelligence; Neurological Impairment; Psychosis). Answers indicative of malingering are summed to obtain a total SIMS score (*α* = .85).

##### Mock crime event

Next, all participants were requested to pay attention to the mock crime video (i.e., approximately 2.30 minutes). Other than a brief dialogue between two characters in the beginning, the mock crime video is mainly accompanied by background music. Participants were asked to pay attention to the mock crime movie and to identify themselves with the character that showed up first on the scene (i.e., the offender). The video contained a violent scene depicting a fight between two armed men. The scene ended with one of the men being strangled by the other. After the video, there was a 10-min interval during which participants performed a filler task (puzzle).

##### Memory test phase (T1)

After completing the filler task, simulator and confessor groups were asked to imagine themselves being arrested on the suspicion of having committed the murder. Additionally, they were told that a witness saw them with the victim, and they were asked to report every piece of information they could remember about the crime. Similar to previous studies, free-recall and cued-recall tests were employed as memory measures^2^ (e.g., Bylin & Christianson, 2002; Mangiulli et al., 2018; Van Oorsouw & Merckelbach, 2004). With respect to the free recall, simulators were instructed to describe the events as if they had great difficulties in remembering what happened in an attempt to evade punishment. By doing so, simulating participants were free to omit, distort, or even introduce distorted or new information about the mock crime. In contrast, confessors, were requested to collaborate with the police and to honestly report as many details as possible about the crime.

Immediately after this free recall, simulators and confessors were asked to answer 14 cued-recall questions about the mock crime event, and they were requested to adhere to the previously given instruction about the role they were playing (i.e., simulating or confessing). Note that controls did not receive any instruction, meaning that they were not tested after being exposed to the mock crime video during the first memory phase. Finally, participants were scheduled for a new session 1 week later.

##### Rehearsal induction

During the week between the presentation of the video clip and the retest phase, a random half of the entire sample (including the three conditions: simulators, confessors, and controls) received frames^3^ of the crime on their smartphones via WhatsApp,^4^ three times in different daytime hours. Nine frames were directly extracted from the video and reported the crucial scenes of the mock crime. A “reminder” of the meeting scheduled for the second experimental memory session followed each frame. Each condition (simulators vs. confessors vs. control) received the same number of cues. The other half of the sample did not receive any reminders during the week.

##### Memory retest phase (T2)

One week after the first memory phase, all participants (simulators vs. confessors vs. controls) returned to the testing room and they were all asked to honestly respond to the free and cued recall tasks. This time, in contrast to the instructions given at T1, simulating participants were requested to describe what they remembered from the mock crime movie, thus giving up to their role as simulators. Confessors again received the instruction to report what they remembered about the crime. Finally, controls were asked to report as many details as possible about the mock crime video they watched 1 week earlier. In the cued-recall task, all participants honestly responded to the same 14 questions as presented 1 week earlier. Once participants finished the second session, they were debriefed and thanked individually.

#### Memory test–retest scoring

##### Free recall

The mock crime video was divided into 50 critical information units. Participants earned one point for every correct unit reported (maximum = 50). A critical information unit was defined as a significant fragment of the video clip relevant for the whole story (e.g., “I had pointed the gun at the back of the victim’s head”). Instead, a partially correct answer was assigned a half point (e.g., “I had pointed the gun at the victim”). The correct score was transformed into proportions (range: 0–1) by dividing the number of information units correctly reported by each respondent by the maximum obtainable score of 50. Additionally, the number of errors was identified (i.e., introduction of new information which was not part of the mock crime event, such as “That day, I was not alone,” or which distorted information, such as “I strangled the victim with my hands”). Free-recall outcomes were scored by the first author and two student assistants who were blind to condition.

##### Cued recall

Fourteen questions pertaining to the mock crime were assessed during a cued-recall task (e.g., presence of weapon and blood, characters’ clothing, details about the location and the surroundings). To calculate cued-recall scores, one point was given for each correct answer (e.g., Question: “With what was the victim was armed?” “He had a weapon and a knife,” while half a point was given for a partially correct answer; e.g., “Yes, he had a weapon”). When participants provided no detail (e.g., “I do not remember”), they received no points. The maximum obtainable score was 14. To obtain a correctness score, proportions (range: 0–1) were calculated by dividing the score obtained in the questionnaire by the maximum obtainable score. Additionally, also for the cued recall, the number of errors was identified (e.g., “The victim had a rifle”).

#### Statistical analysis

Our data were analysed as follow. First, we ran independent-samples *t* tests on both free and cued recalls (i.e., correctness and error scores) to check whether simulating participants complied with the instruction received during the first memory phase (T1). Second, a 3 × 2 between-subjects analysis of variance (ANOVA) was used only on participants’ free and cued recalls at the retest memory phase (T2). Comparisons between groups were performed using analysis of contrasts (Levine, 2013). Where data were not approximately normally distributed, we used the Scheirer–Ray–Hare extension of the Kruskal–Wallis test, and Mann–Whitney *U* test as follow up tests.

### Results

#### Data distribution

A Shapiro–Wilk’s test (*p* > .05; Shapiro & Wilk, 1965) and visual inspection of their histograms, normal Q-Q plots, and box plots indicated that free-recall and cued-recall correctness scores were approximately normally distributed over the three conditions (simulators, *p* = .589 and .632; confessors, *p* = .629 and .218; controls, *p* = .169 and .255, respectively). Yet free-recall and cued-recall error scores were not approximately normally distributed over the three conditions (all *p*s < .05).

#### Manipulation check on simulating participants’ instruction

To verify whether simulators properly followed their instruction during T1, two independent *t* tests were run on both free and cued correctness and error scores. With respect to the free recollection at T1, simulating participants (*M* = .02; 95% CI [.01, .03]) disclosed less correct information than confessors did (*M* = .25; 95% CI [.23, .28]), *t*(58) = −15.75, *p* < .001, 95% CI [−.25, −.20], *d* = 4.46. In addition, participants who were instructed to feign amnesia (*M* = 7.13; 95% CI [6.23, 8.07]) reported more errors than confessors did (*M* = 1.66; 95% CI [.87, 2.72]), *t*(58) = 8.06, *p* < .001, 95% CI [4.10, 6.22], *d* = 2.08.

A similar pattern of results to free recall was also observed for the cued-memory test at T1. Simulators (*M* = .22; 95% CI [.18, .26]) recalled less correct information than confessors did (*M* = .54; 95% CI [.50, .57]), *t*(58) = −11.60, *p* < .001, 95% CI [−.37, −.26], *d* = 2.90. Finally, simulating participants (*M* = 2.73; 95% CI [2.16, 3.36]) reported more errors than confessors did (*M* = 1.53; 95% CI [1.18, 1.89]), *t*(58) = 3.26, *p* = .002, 95% CI [.46, 1.93], *d* = .84.

#### Correctness score

**Free recall.** Free-recall correctness scores were entered in a 3 (condition: simulators vs. confessors vs. controls) × 2 (rehearsal induction: reminder vs. no reminder) between-subjects ANOVA, conducted on the retest memory phase (T2). While the main effect of condition was found to be significant *F*(2, 83) = 17.74, *p* < .001, η_p_^2^ = .30, the main effect of rehearsal was not, *F*(1, 83) = 1.82, *p* = .18, η_p_^2^ = .02. The condition × rehearsal interaction was also found to be significant, *F*(2, 83) = 3.36, *p* = .03, η_p_^2^ = .07. This interaction revealed that, among participants who were not subjected to the rehearsal induction, no significant differences were observed between simulators and confessors in contrast with our hypothesis (Hypothesis 1), *p* = .65, 95% CI [-.06, .04], η_p_^2^ = .00. However, both simulators and confessors outperformed controls, *p* = .015, 95% CI [.01, .12], η_p_^2^ = .07, and *p* = .004, 95% CI [.02, .13], η_p_^2^ = .09, respectively.

**Fig. 1:**
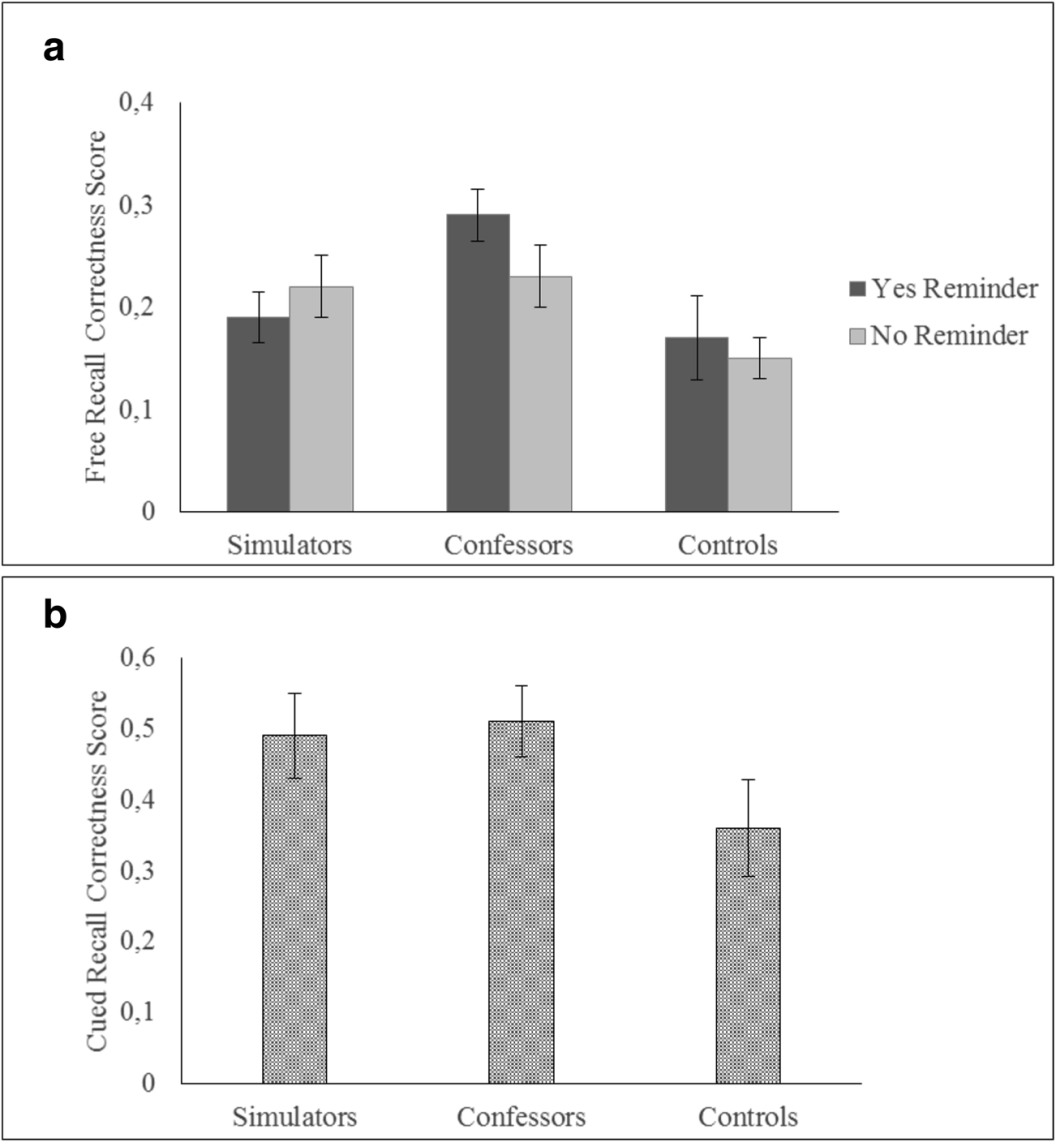
Free and cued recall correctness scores at T2. **a** The condition × rehearsal induction interaction. **b** The main effect of condition. Error bars represent 95% confidence intervals

Among participants who were given reminders via WhatsApp, simulators reported less correct information than confessors against our prediction (Hypothesis 2), *p* < .001, 95% CI [-.14, -.04], η_p_^2^ = .15. Yet, partially supporting our hypothesis (Hypothesis 3), only confessors reported more correct details than controls did, *p* < .001, 95% CI [.06, .16], η_p_^2^ = .20, while this latter group did not differ from simulators, *p* = .43, 95% CI [.02, .06], η_p_^2^ = .00. Thus, only confessors benefitted from the rehearsal induction as compared with the control group. Data for the free recall are displayed in Fig. 1a.

##### Cued recall

For the cued-recall correctness scores, identical analyses to the free recall were run on the retest memory phase (T2). Only the main effect of condition reached the significance, *F*(2, 83) = 12.29, *p* < .001, η_p_^2^ = .22. No other main or interaction effects were observed, *F*s(1, 83) < 1.15, *p* > .28. Overall, while no significant differences were found between simulators and confessors at T2, *p* = .61, 95% CI [-.09, .06], η_p_^2^ = .00, both those groups outperformed controls at T2, *p* = .001, 95% CI [.05, .21], η_p_^2^ = .11, and *p* = .004, 95% CI [.06, .22], η_p_^2^ = .10, respectively. Data for the cued recall are shown in Fig. 1b. Also see Table S1 in the Supplemental Materials for cued recall descriptive.

#### Error scores

##### Free recall

Free-recall error scores were analysed by using a Scheirer–Ray–Hare extension of the Kruskal–Wallis test. Neither main nor interaction effects reached statistical significance, all *H*s < 9.65, *p*s > .13. This indicated that, against our prediction (Hypothesis 4), overall simulators (*Mdn* = 1.50) did not report more errors than confessors did (*Mdn* = 1.00) regardless of the rehearsal induction, *U* = 354.00, *p* = .141, *r* = .19. For completeness sake, participants’ means and confidence intervals from free-recall error scores are reported in Table S3 in the Supplemental Materials.

##### Cued recall

A Scheirer–Ray–Hare extension of the Kruskal–Wallis test ran on participants’ cued-recall error scores revealed that neither main nor interaction effects reached statistical significance, all *H*s < 15.50, *p*s > .11. Hence, against our prediction (Hypothesis 4), simulators (*Mdn* = 1.00) did not differ from confessors (*Mdn* = 1.00) in the number of cued-recall errors reported at T2 irrespective of the rehearsal induction, *U* = 411.50, *p* = .547, *r* = .07. Participants’ means and confidence intervals from cued recall error scores are shown in Table S3 in the Supplemental Materials.

#### Exploratory analysis on simulating participants’ free-recall reports

To stress the potential effects of simulating amnesia on genuine memory for a mock crime, simulating participants’ free recall reports were analysed (i.e., *N* = 15, no-reminder condition). We explored how much of the same information was recollected across both memory tests. On average simulators recalled at T2 63% of the correct information they also mentioned during T1. Simulating participants mostly reported in both recalls the crime scene (87%), physical fight between them and the victim (33%), escaping after the criminal act (33%), and presence of weapons (20%).

### Discussion

At the baseline condition (i.e., no reminder), we did not observe any difference between simulators and confessors with respect to either the number of correct details or errors reported, both on the free-recall and cued-recall tests. Although individuals usually report more correct information in open-ended cued-recall tests than in free-recall tests (Craik & McDowd, 1987; Padilla-Walker & Poole, 2002), those results are in contrast with previous research that found evidence for a memory-undermining effect of simulated amnesia for a crime (e.g., Bylin & Christianson, 2002; Christianson & Bylin, 1999; Mangiulli et al., 2018; Van Oorsouw & Merckelbach, 2004, 2006). It should be noted here that despite using a sizeable initial sample, the number of participants left after the rehearsal manipulation (i.e., *N* = 15) might have led to underpowered findings. Hence, data from the pilot restrict us from providing a firm conclusion pertaining to why we did not replicate findings from previous research (e.g., Christianson & Bylin, 1999; Mangiulli et al., 2018; Van Oorsouw & Merckelbach, 2004, 2006).

Most important, and in line with more recent studies on crime-related amnesia (e.g., Mangiulli et al., 2018; McWilliams, Goodman, Lyons, Newton, & Avila-Mora, 2014), simulators overall recalled more correct information than controls did, thereby supporting the idea that some rehearsal might be present at T1 when simulators are asked to recall the crime while role-playing amnesia. That is, even while feigning memory problems, simulators might have rehearsed the actual crime while attempting to provide a convincing amnesia story (e.g., Mangiulli et al., 2018; McWilliams et al., 2014). It is reasonable that an actual rehearsal occurred while simulators were tested in the first memory test phase and not by mock crime frames sent via WhatsApp.

Lastly, there are no good reasons for why reminders did not have a beneficial effect (i.e., more correct recollection and less memory errors) on simulators’ memory performance as compared with confessors’ as predicted. Perhaps, confessors’ memory might have been triggered by the frames to a greater extent than simulators’ memory. Conceivably, the way in which both groups engaged with the rehearsal induction had a crucial role in participants’ memory improvement or lack thereof. It remains speculative, however, why confessors benefitted from reminders and simulators did not. Note that we do not know whether participants actually looked at frames and rehearsed them or they just ignored the reminders. And even if participants checked frames on their smartphones, it might be doubtful, considering this as a form of actual rehearsal.

## Study 2

In Study 2, we used the same procedure of Study 1 (pilot). However, because the pilot study might have been underpowered, and we did not have control over the rehearsal induction, we changed the pilot in two ways. First, a larger sample of participants was recruited. Second, we adopted a different rehearsal induction modality. That is, we instructed participants to rehearse the mock crime by providing them with written sequences of the crime stimulus. In line with Study 1, we anticipated simulating amnesia would undermine memory (Hypothesis 1), but because of the rehearsal induction, simulators would perform at a similar level as confessors (Hypothesis 2). We again predicted both simulators and confessors would remember more correct crime information than controls (Hypothesis 3). Finally, we expected that simulators would commit more errors than confessors irrespective of the rehearsal manipulation (Hypothesis 4).

### Method

#### Participants and design

In this second study, 150 undergraduate students (83% women; *M*_age_ = 19.95 years, *SD* = 2.52) from the University of Bari were recruited using advertisements and a snowballing sampling technique (Goodman, 1961). Each participant received a course credit hour for taking part in the study. Participants were randomly assigned to the experimental condition—simulators (*N* = 50), confessors (*N* = 50), and controls (*N* = 50), divided over reminder versus no-reminder conditions. This study used a 3 (condition: simulators vs. confessors vs. controls) × 2 (rehearsal induction: reminder vs. no reminder) between-subjects design. The dependent variables were identical to those in Study 1.

#### Measures and procedure

The materials and measures were the same as those used in Study 1.^5^ Only the rehearsal induction modality differed from that adopted in Study 1. First, we transcribed the whole video, dividing it into 18 sentences. These sentences were put together on a table in a wrong temporal order. We asked half of participants (simulators vs. confessors vs. controls) to put these sentences in the right order from the first temporal event to the last. This manipulation was repeated twice during the week between the memory test (T1) and retest (T2) phases by asking participants to come to the lab to complete the task. Similar to Study 1, participants in the control condition were not provided with any instructions after being exposed to the crime video, meaning that their memory performance was tested only during the memory retest phase (T2).

Both free-recall and cued-recall scores were transformed into proportions. Additionally, the number of errors was identified. Free-recall and cued-recall outcomes were scored by the first author and by two student assistants. Finally, the same statistical approach of Study 1 was adopted.

### Results

#### Data distribution

A Shapiro–Wilk’s test (*p* > .05; Shapiro & Wilk, 1965) and visual inspection of their histograms, normal Q-Q plots, and box plots indicated that free-recall and cued-recall scores were approximately normally distributed over the three conditions (simulators, *p* = .357 and .197; confessors, *p* = .133 and .582; control, *p* = .470 and .777, respectively). Yet data were not approximately normally distributed over the three conditions with respect to both free and cued recall error scores (all *p*s < .05).

#### Manipulation check on simulating participants’ instruction

Two independent *t* tests were run on both free and cued correctness and error scores to check whether simulating participants adhered to the instruction given during T1. With respect to the free recollection at T1, as expected simulators (*M* = .03; 95% CI [.02, .04]) recalled less correct information than confessors did (*M* = .22; 95% CI [.19, .24]), *t*(98) = −14.19, *p* < .001, 95% CI [−.21, −.16], *d* = 3.14. Moreover, simulating participants (*M* = 10.63; 95% CI [7.70, 14.16]) reported more errors than confessors did (*M* = 4.52; 95% CI [3.22, 6.24]), *t*(98) = 3.37, *p* = .001, 95% CI [2.51 9.70], *d* = .68.

Results from cued memory test were similar to those depicted above. Simulating participants (*M* = .26; 95% CI [.22, .30]) disclosed less correct information than confessors did (*M* = .57; 95% CI [.53, .60]), *t*(98) = −11.85, *p* < .001, 95% CI [−.35, −.25], *d* = 2.46. However, no differences were found between simulators (*M* = 4.90; 95% CI [4.17, 5.63]) and confessors (*M* = 4.60; 95% CI [4.16, 5.07]) on cued recall error scores, *t*(98) = .66, *p* = .506, 95% CI [−.59, 1.19], *d* = .13.

#### Correctness scores

##### Free recall

**Fig. 2:**
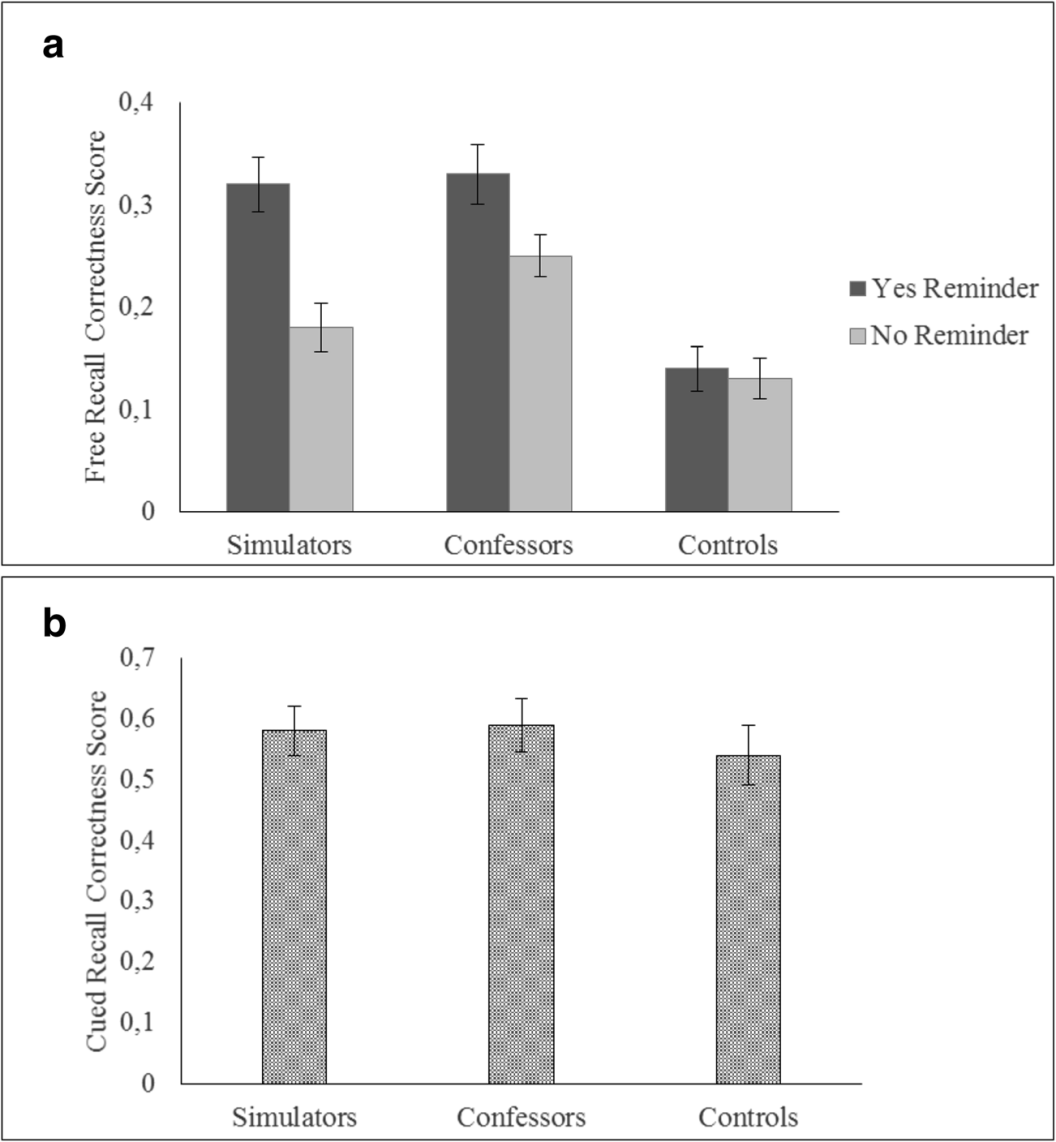
Free and cued recall correctness scores at T2. **a** The condition × rehearsal induction interaction. **b** The main effect of condition. Error bars represent 95% confidence intervals

Free-recall correctness scores were analysed using a 3 (condition: simulators vs. confessors vs. control) × 2 (rehearsal induction: reminder vs. no reminder) between-subjects ANOVA only for the retest memory phase (T2). Significant effects of condition and rehearsal induction were found, *F*(2, 144) = 86.56, *p* < .001, η_p_^2^ = .54, and, *F*(1, 144) = 60.43, *p* < .001, η_p_^2^ = .29, respectively. These main effects were qualified by a significant condition × rehearsal induction interaction, *F*(2, 144) = 10.54, *p* < .001, η_p_^2^ = .13. That is, confessors remembered more correct information than simulators when both groups did not get reminders, thereby indicating the standard memory-undermining effect of simulating amnesia (Hypothesis 1), *p* = .008, 95% CI [.12.01], η_p_^2^ = .03. Yet, in line with our prediction (Hypothesis 2), no significant difference was found at T2 between simulators and confessors who were subjected to rehearsal induction, *p* = .639, 95% CI [-.05 .02], η_p_^2^ = .00. Additionally, both simulators and confessors reported more correct information than controls did (Hypothesis 3), irrespective of being subjected to the rehearsal induction, *p* < .001, 95% CI [.12 .21], η_p_^2^ = .27, and *p* < .001, 95% CI [.13 .22], η_p_^2^ = .29, or not, *p* = .02, 95% CI [.01 .11], η_p_^2^ = .03, and *p* < .001, 95% CI [.07 .18], η_p_^2^ = .14, respectively. Data for the free recall are shown in Fig. 2a.

**Cued recall.** On correctness cued-recall scores, identical analyses to the free recall were run. Main effects of condition and rehearsal induction were significant, *F*(2,144) = 17.80, *p* < .001, η_p_^2^ = .20, and *F*(1, 144) = 22.42, *p* < .001, η_p_^2^ = .13, respectively. Yet, no interaction effect was found, *F*(2, 144) = .05, *p* = .94. As in Study 1, while no differences were observed between simulators and confessors, *p* = 1.00, 95% CI [-.06 .04], η_p_^2^ = .00, both groups outperformed controls, *p* < .001, 95% CI [.05 .16], η_p_^2^ = .16, and *p* < .001, 95% CI [.06 .17], η_p_^2^ = .29, respectively. Lastly, individuals who were involved in the rehearsal manipulation (*M* = . 59; 95% CI [.56, .61]) overall recalled more correct information than those who were not (*M* = .50; 95% CI [.48, .53]), *p* < .001, 95% CI [.04 .12], η_p_^2^ = .11. Data for the cued recall are displayed in Fig. 2b. See Table S2 in the Supplemental Materials for cued recall descriptive items.

#### Error scores

##### Free recall

Free-recall error scores were analysed by using a Scheirer–Ray–Hare extension of the Kruskal–Wallis test. Only the main effect of condition was found statistically significant, *H* = 11.84, *p* = .002, while neither other main nor interaction effects were not, *H*s < 22.18, *p*s > .22. This analysis showed that, against our expectation (Hypothesis 4), no difference between simulators (*Mdn* = 2.00) and confessors (*Mdn* = 1.00) was observed in the number of errors reported irrespective of the rehearsal manipulation, *U* = 1105.00, *p* = .310, *r* = .10. For completeness sake, all participants’ means and confidence intervals from free-recall error scores are reported in Table S4 in the Supplemental Materials.

##### Cued recall

With respect to the cued-recall error scores, a Scheirer–Ray–Hare extension of the Kruskal–Wallis test revealed a main effect of condition, *H* = 14.94, *p* < .001, and a condition × rehearsal induction interaction effect, *H* = 38.96, *p* = .002. The main effect of rehearsal did not reach statistical significance, *H* = 14.78, *p* = .097. Interestingly, contradicting our hypothesis (Hypothesis 4), among those who received the reminders, simulators (*Mdn* = 2.00) made fewer errors than confessors did (*Mdn* = 3.00), *U* = 177.00, *p* = .007, *r* = .26. However, no significant differences were found between these two groups (*Mdn*_simulators_ = 4.00; *Mdn*_confessors_ = 4.00) when participants were not subjected to the rehearsal induction, *U* = 245.00, *p* = .182, *r* = .13. All participants’ means and confidence intervals from cued recall error scores are displayed in Table S4 in the Supplemental Materials.

#### Exploratory analysis on simulating participants’ free-recall reports

In line with Study 1, simulating participants’ free recall reports at T1 were analysed (i.e., *N* = 25, no-reminder condition) to further explore the simulating amnesia effect on individuals’ memory for a crime. At T2, simulators repeated 52.96% of the correct information they had also reported at T1. Specifically, simulating participants mostly recalled in both memory test phases the crime scene (60%), physical fight between them and the victim (28%), presence of weapons (24%), and what the victim wore (20%).

### Discussion

Contrary to Study 1, when simulators were not encouraged to rehearse the crime, feigning amnesia had a detrimental effect on memory (i.e., when simulators were compared with those who confessed to the crime at T2). On the other hand, when engaging in the rehearsal induction “task” of Study 2, simulating participants no longer showed a memory deficit for the mock crime when they had to give up their role as a simulator as compared with confessors. Although the number of errors did not decrease when simulators received reminders, the rehearsal induction adopted in Study 2 seemed to help simulators to protect their memory from decay and to perform at a similar level as people who told the truth repeatedly. That is, while in Study 1 participants might have passively looked at the frames of the mock crime video sent via WhatsApp, it is likely that in Study 2 they more actively interacted with the reminders provided.

Finally, even without memory reminders, simulators still outperformed controls, in line with Study 1. This might indicate that the mere act of telling a simulated amnesia story at T1 could perhaps act as a form of rehearsal.

## General discussion

We sought to examine whether simulators who were prompted with cues provided in two different modalities would keep relevant information of the (mock) criminal experience. Only the rehearsal induction in Study 2 was successful in doing so for ex-simulators (see Table 1). Simulators involved in the rehearsal induction did not forget the crime even though they previously simulated amnesia. We showed that rehearsal can promote memory for the crime of former simulators and may return their mnemonic capacities to the level of a person who was interviewed repeatedly and honestly accounted for the experience. This might suggest that, to some extent, reminders might serve as a memory-enhancement technique when individuals are actively involved in the criminal experience—by being the offender—rather than when they are simply bystanders. For that reason, one could question whether the difference between simulators and truth-tellers (i.e., confessors) should be seen as a “memory-undermining effect” due to claims of simulated crime-related amnesia.

**Table 1:** Summary of main findings pertaining to correctness scores in Study 1 (pilot) and Study 2

	Free recall				Cued recall	
	Simulators vs. confessors		Simulators vs. controls		Simulators vs. confessors	Simulators vs. controls
Reminder	*Yes*	*No*	*Yes*	*No*	*Overall*	*Overall*
**Study 1**	<**	ns	ns	>*	ns	>**
**Study 2**	ns	<*	>**	>*	ns	>**

*Note.* “<” and “>” indicates simulators having lower and higher correctness scores, respectively. “*” and “**” expresses *p* < .05 and *p* < .001, respectively. Finally, “ns” refers to nonsignificant findings. Because for the cued recall there was no Condition × Rehearsal Induction interaction effect, main findings are displayed collapsed (i.e., *overall*)

Moreover, although to some degree, simulating amnesia might jeopardize their memories, offenders who pretend to suffer from memory loss will still recollect more crime-related information than those who are not initially interviewed (i.e., controls). In both of our studies, indeed, simulators outperformed controls irrespective of the rehearsal induction (see Table 1). This pattern of findings is in contrast with previous research (e.g., Christianson & Bylin, 1999; Sun et al., 2009; Van Oorsouw & Merckelbach, 2004, 2006). Arguably, simulators could have encoded more crime-related information than controls simply because they were asked to identify themselves with the offender (e.g., active task-participation in memory; Nahari & Ben-Shakhar, 2011). Alternatively, during the first memory phase, simulating amnesia might indirectly represent a way to rethink of the crime so that it generated a significant rehearse-practice effect. This also entails that feigning amnesia in itself requires active cognitive processing (e.g., Mangiulli et al., 2018; McWilliams et al., 2014), thereby leading simulators to retain some traces of the crime more efficiently than controls. Indeed, one could argue that simulating amnesia by distorting or self-generating new information might undermine memory for a crime less strongly than simulating amnesia by omitting information might do. Germane to this, Mangiulli, Van Oorsouw, Curci, and Jelicic (2019) recently showed that when simulators are specifically asked to omit crucial crime-related details (e.g., how the crime occurred) they exhibit a significantly weaker recollection for those items than for other minor information (e.g., location of the crime). On the other hand, when simulators are not instructed how to simulate memory loss for a crime, simulating amnesia might act as a buffer against forgetting (e.g., Mangiulli et al., 2019). In the current studies, however, we did not ask simulators which strategy they had used in an attempt to feign amnesia for a crime. For that reason, it would be interesting to further investigate possible strategies adopted by simulators in future studies.

Several caveats of our studies need to be addressed. First, both our samples principally consisted of undergraduates students, which differ on so many levels from individuals who commit severe crimes (Schacter, 1986) . In real-life circumstances, criminal experiences often have a strong emotional component that undoubtedly might affect perpetrators memory for a crime. Second, our findings suggest little about the actual impact that reminders might have played on participants’ memory performance. Namely, our data appear to indicate that *any* type of reminder might boost simulators’ genuine memory for a crime when a memory-undermining effect of feigning amnesia occurs in the first place. However, based on the current findings, it is difficult to establish to which extent reminders of the crime mitigated such detrimental memory effect. Therefore, future studies should test for effects of reminders on memory impairment due to simulated amnesia. Also, we cannot rule out that other factors might have influenced overall participants’ memory performance, such as active versus passive individuals’ role towards the reminders, the number of repetition adopted as rehearsal induction, and the nature of the cues (i.e., images vs. words). One could even argue that type of reminder would affect correct recollection differently—based on the memory test adopted to measure the final recollection. Because individuals often are better at reporting events when prompted by open-ended questions than being asked to freely account for them (Craik & McDowd, 1987; Padilla-Walker & Poole, 2002), one might predict that reminders would have a minimum, if not null, effect when using a cued recall test to elicit the experience. Therefore, future research may shed new light on the possible consequences of reminders on simulators’ actual memory for a crime by taking into consideration those factors.

In closing, given the possibility of overinterpreting both null and significant effects with small samples, our results should be treated with caution. However, if it is true that rehearsal effects increase with the number of rehearsals (Bergman & Roediger, 1999), it would be perhaps possible to use mnemonic elicitation techniques such as cognitive cues to stimulate offenders to rehearse their criminal actions during information-gathering investigations (e.g., Meissner, Redlich, Bhatt, & Brandon, 2012; Memon, Meissner, & Faser, 2010). Take, for instance, plea bargaining situations wherein perpetrators who have previously simulated amnesia may not properly remember the criminal act—not because they have previously simulated amnesia for this information, but because they have not been sufficiently encouraged to rehearse the event. Given the importance of disclosing crime-related information in high-stake cases, the results of the current studies might be used to find a way to increase offenders’ possibility to rehearse the criminal experiences during police interviews.

## Electronic supplementary material

ESM 1 (DOCX 87 kb)

## Footnotes

1 The SIMS (Smith & Burger, 1997) includes five subscales which were not directly relevant for our purposes. None of our participants (simulators vs. confessors vs. controls) was excluded from our study based on their SIMS results. The conditions did not differ in their malingering tendency before the memory test phase (*p* > .05).

2 The measures and materials used in both studies, including the mock crime video, were in Italian.

3 The messages were randomly sent to participants. Neither particular sampling design nor specifics criteria were followed.

4 WhatsApp Messenger is a freeware, cross-platform messaging and voice-over IP (VoIP) service (https://www.whatsapp.com/). It allows users to send, amongst different media, text messages and pictures.

5 As in Study 1, none of our participants (simulators vs. confessors vs. controls) was excluded based on their SIMS findings. Furthermore, no differences in malingering tendency were found between participants of the different groups before the memory test phase (*p* > .05).
