# Supplementary material for: Do reminders of the crime reverse the memory-undermining effect of simulating amnesia?
Source: Mem Cognit. 2019 May 17;47(7):1375–85. doi: 10.3758/s13421-019-00939-z (PMC6800869; doi:10.3758/s13421-019-00939-z)
Supplement: Supplementary file 1 — (DOCX 87 kb) [file 13421_2019_939_MOESM1_ESM.docx]

**Supplemental Materials**

| **Table 1.** Descriptive items for cued recall correctness scores reported by each condition split by reminder vs. no reminder at T2 (study 1). | | | | | | | | | | | | |
| --- | --- | --- | --- | --- | --- | --- | --- | --- | --- | --- | --- | --- |
|  | Conditions | | | | | | | | | | | |
|  | Simulators (*N* = 30) | | | | Confessors (*N* = 30) | | | | Controls (*N* = 30) | | | |
| Reminder | Yes | | No | | Yes | | No | | Yes | | No | |
|  | *M* | *95%CI* | *M* | *95%CI* | *M* | *95%CI* | *M* | *95%CI* | *M* | *95%CI* | *M* | *95%CI* |
| Items |  | | | | | | | | | | | |
| 1. Where did the murder take place? | .75 | [.60 .89] | .80 | [.63 .93] | .75 | [.50 .91] | .63 | [.43 .83] | .76 | [.57 .92] | .53 | [.30 .73] |
| 2. How was the victim dressed? | .28 | [.17 .42] | .23 | [.10 .36] | .25 | [.12 .37] | .16 | [.06 .30] | .26 | [.11 .46] | .03 | [.00 .11] |
| 3. How many gunshots were fired? | .14 | [.00 .36] | .17 | [.00 .37] | .29 | [.08 .54] | .30 | [.10 .53] | .15 | [.00 .38] | .15 | [.00 .38] |
| 4. What colour the wall tiles were? | .57 | [.29 .86] | .73 | [.47 .93] | .58 | [.33 .83] | .73 | [.53 .93] | .62 | [.38 .85] | .46 | [.23 .69] |
| 5. Was there blood on the crime scene? | .68 | [.57 .79] | .60 | [.47 .73] | .71 | [.58 .83] | .83 | [.70 .93] | .58 | [.42 .73] | .50 | [.35 .65] |
| 6. Was the victim armed? | .82 | [.61 1] | 1 | [1 1] | .83 | [.58 1] | .93 | [.80 1] | .85 | [.62 1] | .85 | [.62 1] |
| 7. Which weapon was used to kill the victim? | .75 | [.50 .93] | .80 | [.60 1] | 1 | [1 1] | .80 | [.60 1] | .46 | [.23 .69] | .54 | [.23 .77] |
| 8. Were you armed? | .93 | [.79 1] | .80 | [.60 1] | 1 | [1 1] | .87 | [.67 .1] | .81 | [.58 1] | .54 | [.31 .81] |
| 9. Was there any object on the crime scene? | .25 | [.11 .39] | .23 | [.07 .40] | .29 | [.17 .42] | .13 | [.03 .27] | .04 | [.00 .12] | 0 | [0 0] |
| 10. Did the victim wear earrings? | .14 | [.00 .36] | .37 | [.13 .63] | .50 | [.25 .75] | .13 | [.00 .33] | .23 | [.00 .46] | .15 | [.00 .38] |
| 11. Were you and the victim alone on the crime scene? | 1 | [1 1] | .93 | [.80 1] | 1 | [1 1] | 1 | [1 1] | 1 | [1 1] | 1 | [1 1] |
| 12. How were you dressed? | .57 | [.36 .79] | .53 | [.33 .73] | .50 | [.29 .71] | .33 | [.17 .53] | .19 | [.00 .42] | .15 | [.00 .35] |
| 13. Were there fire extinguishers on the crime scene? | .61 | [.36 .86] | .60 | [.33 .87] | .33 | [.08 .58] | .47 | [.20 .73] | .27 | [.08 .54] | .38 | [.15 .69] |
| 14. Did the victim get immediately killed? | .54 | [.29 .79] | .20 | [.00 .40] | .75 | [.50 1] | .47 | [.20 .73] | .31 | [.08 .54] | .38 | [.15 .69] |
| *Note*: Means and 95%Coenfidence Intervals are displayed in absolute numbers (range: 0-1). | | | | | | | | | | | | |

| **Table 2.** Descriptive items for cued recall correctness scores reported by each condition split by reminder vs. no reminder at T2 (study 2). | | | | | | | | | | | | |
| --- | --- | --- | --- | --- | --- | --- | --- | --- | --- | --- | --- | --- |
|  | Conditions | | | | | | | | | | | |
|  | Simulators (*N* = 50) | | | | Confessors (*N* = 50) | | | | Controls (*N* = 50) | | | |
| Reminder | Yes | | No | | Yes | | No | | Yes | | No | |
|  | *M* | *95%CI* | *M* | *95%CI* | *M* | *95%CI* | *M* | *95%CI* | *M* | *95%CI* | *M* | *95%CI* |
| Items |  | | | | | | | | | | | |
| 1. Where did the murder take place? | .82 | [.66 .96] | .52 | [.36 .68] | .72 | [.56 .88] | .64 | [.44 .80] | .66 | [.50 .82] | .78 | [.62 .92] |
| 2. How was the victim dressed? | .30 | [.18 .40] | .10 | [.02 .20] | .32 | [.20 .42] | .12 | [.04 .20] | .20 | [.08 .32] | .18 | [.08 .28] |
| 3. How many gunshots were fired? | .92 | [.80 1] | .24 | [.08 .40] | .82 | [.08 .54] | .32 | [.16 .52] | .12 | [.00 .24] | .04 | [.00 .12] |
| 4. What colour the wall tiles were? | .86 | [.70 .98] | .78 | [.60 .92] | .66 | [.46 .82] | .76 | [.60 .92] | .70 | [.52 .88] | .72 | [.52 .88] |
| 5. Was there blood on the crime scene? | .68 | [.58 .78] | .64 | [.56 .72] | .76 | [.64 .86] | .58 | [.52 .66] | .64 | [.54 .76] | .58 | [.52 .66] |
| 6. Was the victim armed? | 1 | [1 1] | .92 | [.80 1] | .98 | [.94 1] | 1 | [1 1] | .82 | [.68 96] | .88 | [.76 1] |
| 7. Which weapon was used to kill the victim? | .98 | [.94 1] | .88 | [.72 1] | 1 | [1 1] | .96 | [.88 1] | .64 | [.44.80] | .70 | [.52 .86] |
| 8. Were you armed? | .92 | [.80 1] | .88 | [.72 1] | 1 | [1 1] | .88 | [.76 1] | .88 | [.76 1] | .72 | [.52 .88] |
| 9. Was there any object on the crime scene? | .14 | [.12 .42] | .12 | [.04 .21] | .16 | [.06 .28] | .10 | [.02 .18] | .10 | [.00 .18] | .06 | [.00 .12] |
| 10. Did the victim wear earrings? | .26 | [.12 .42] | .20 | [.04 .36] | .24 | [.08 .40] | .06 | [.00 .16] | .20 | [.04 .36] | .22 | [.08 .40] |
| 11. Were you and the victim alone on the crime scene? | .96 | [.88 1] | 1 | [1 1] | .98 | [.94 1] | 1 | [1 1] | 1 | [1 1] | 1 | [1 1] |
| 12. How were you dressed? | .32 | [.22 .40] | .32 | [.18 .46] | .50 | [.34 .66] | .36 | [.24 .50] | .28 | [.16 .42] | .28 | [.14 .44] |
| 13. Were there fire extinguishers on the crime scene? | .44 | [.24 .64] | .40 | [.20 .60] | .38 | [.20 .56] | .40 | [.20 .60] | .36 | [.20 .56] | .44 | [.24 .64] |
| 14. Did the victim get immediately killed? | .12 | [.00 289] | .52 | [.32 .72] | .40 | [.20 .60] | .46 | [.28 .66] | .56 | [.36 .76] | .26 | [.10 .44] |
| *Note*: Means and 95%Coenfidence Intervals are displayed in absolute numbers (range: 0-1). | | | | | | | | | | | | |

| **Table 3**. Free and cued recall error scores for each condition during the retest memory phase (T2; Study 1). | | | | | | | |
| --- | --- | --- | --- | --- | --- | --- | --- |
| **Free Recall** | | | | | | | |
|  | Simulators | | | Confessors | | Controls | |
| Reminder | Yes | No | | Yes | No | Yes | No |
| Errors | 2.13  [1.28 2.98] | 1.26  [.41 2.11] | | 1.26  [.42 2.15] | 1.93  [1.08 2.78] | 1.86  [1.01 2.71] | 1.00  [.12 1.87] |
| **Cued Recall** | | | | | | | |
|  | Simulators | | | Confessors | | Controls | |
| Reminder | Yes | | No | Yes | No | Yes | No |
| Errors | 2.06  [1.50 2.63] | | .86  [.30 1.43] | 1.26  [.70 1.83] | 1.20  [.63 1.76] | 2.00  [1.43 2.56] | 1.92  [1.34 2.51] |
| *Note*: Table 3 displays proportion errors split by rehearsal induction (reminder vs. no reminder). Errors are reported in absolute numbers. *95%Coenfidence Intervals* are shown between parentheses. | | | | | | | |

| **Table 4**. Free and cued recall error scores for each condition during the retest memory phase (T2; Study 2). | | | | | | |
| --- | --- | --- | --- | --- | --- | --- |
| **Free Recall** | | | | | | |
|  | Simulators | | Confessors | | Controls | |
| Reminder | Yes | No | Yes | No | Yes | No |
| Errors | 2.00  [.77 3.22] | 3.48  [2.25 4.70] | 2.92  [1.69 4.18] | 1.84  [.61 3.06] | 4.30  [3.07 5.52] | 3.32  [2.09 4.54] |
| **Cued Recall** | | | | | | |
|  | Simulators | | Confessors | | Controls | |
| Reminder | Yes | No | Yes | No | Yes | No |
| Errors | 2.34  [1.71 2.97] | 3.72  [3.09 4.35] | 3.60  [2.97 4.23] | 4.44  [3.81 5.07] | 4.68  [4.05 5.31] | 4.08  [3.45 4.71] |
| *Note*: Table 4 shows proportions split by rehearsal induction (reminder vs. no reminder). Errors are reported in absolute numbers. 95%Coenfidence Intervals are shown between parentheses. | | | | | | |
